# Am I seeing things through the eyes of patients? An exercise in bolstering patient attentiveness and empathy

**DOI:** 10.1186/s12913-018-3681-x

**Published:** 2018-12-14

**Authors:** James K. Elrod, John L. Fortenberry

**Affiliations:** 1 Willis-Knighton Health System, 2600 Greenwood Road, Shreveport, LA 71103 USA; 2 LSU Shreveport, 1 University Place, Shreveport, LA 71115 USA

**Keywords:** Customer service, Patient relations, Marketing, Hospitals, Healthcare

## Abstract

**Background:**

Modern marketing thought heavily emphasizes the need for healthcare providers to possess a customer orientation, placing patients at the focal point of attention within health and medical establishments. This has motivated significant investments in tools and techniques that foster outstanding service, attention, and support. Such investments in isolation, however, offer no guarantees that a true customer orientation will emerge. Proper implementation also is required—and that falls on the shoulders of health and medical personnel.

**Discussion:**

The most innovative and expensive of customer-oriented tools and techniques mean very little unless they are placed in the hands of capable individuals possessing the ability and desire to serve patients well. But the rigors of industry life complicate matters, resulting occasionally in lost focus, compromising the patient experience. One of the simplest and most effective methods for encouraging patient attentiveness rests with a reflective exercise that encourages staff members to see themselves and their actions from the perspective of patients. Asking the operative question, “Am I seeing things through the eyes of patients?” serves as an effective reminder of priorities, building empathy and motivating personnel to continually deliver their very best.

**Conclusions:**

Viewing one’s actions from the perspective of patients can be very revealing, opening eyes wide and permitting opportunities for any necessary improvements, making for a simple but powerful learning experience. The “Am I seeing things through the eyes of patients?” reflective exercise helps well-intentioned staff members avoid tendencies which can lull them into states of complacency, ensuring that they remain focused on those in their care.

## Background

Modern marketing thought heavily emphasizes the need for healthcare providers to possess a customer orientation, placing patients at the focal point of attention within health and medical establishments [1–3]. Institutional decisions regarding all facets of service and support are made with patients at the forefront of thought, with intensive efforts being directed toward meeting and exceeding their wants and needs, all while treating them with empathy and respect [4–6]. It represents the exemplification of customer service, attention, and support [3]. In the discipline of marketing, this particular philosophy is known as the marketing concept [3, 7, 8]. As relevant today as ever, it emerged in the 1960s in tandem with the disciplinary subcomponent of consumer behavior which directed concerted attention toward understanding customers and their patronage habits and practices. Subsequent research confirmed the value of customer-focused attention and support, unseating prior philosophies which tended to focus predominantly on institutional capabilities and proficiencies, typically at the expense of served audiences and their defined wants and needs [3, 9, 10].

The importance of healthcare organizations being highly attentive to patients continues in present day, evidenced by extensive institutional investments in assets aimed at meeting and exceeding the desires of target audiences, including innovative technologies, attractive and inviting service environments, wide-ranging service arrays, and similar resources [1, 9, 11, 12]. Further evidence of the value of embracing a customer orientation is found in the wide array of techniques supporting associated advancements, illustrated by robust initiatives in the areas of patient satisfaction (i.e., efforts directed toward meeting and exceeding the wants and needs of care recipients), customer relationship management (i.e., efforts directed toward the delivery of personalized attention, service, and support to patients for purposes of building loyalty), customer experience management (i.e., efforts directed toward crafting all-encompassing experiences that engage patients), and related pursuits [3, 9, 10]. Such institutional efforts are supported and advanced by a burgeoning body of knowledge, with publications in both scholarly (e.g., [13–16]) and trade (e.g., [17–20]) realms directing significant attention toward understanding patients, permitting healthcare providers to more proficiently deliver compelling service experiences.

For those healthcare organizations that have made prudent investments in proper tools (e.g., infrastructure, technologies) and techniques (e.g., programs, policies, procedures), achieving a customer orientation becomes a distinct possibility. Such investments in isolation, however, offer no guarantees that a patient-focused organization will emerge. Proper implementation also is required—and that falls on the shoulders of health and medical personnel. To help ensure that staff members consistently demonstrate patient attentiveness and empathy in their work efforts, Willis-Knighton Health System suggests a simple exercise, one which encourages them to see themselves and their actions from the eyes of patients.

## Discussion

The most innovative and expensive of customer-oriented tools and techniques mean very little unless they are placed in the hands of capable individuals possessing the ability and desire to serve patients well. Hiring right is essential but the realities of industry life complicate matters even when workforces teem with capable and devoted personnel. In modern healthcare establishments, demands are significant, obligations are ever-increasing, staffing shortages are common, and shifts can be very lengthy and tiring, making even the most dedicated staff members weary. Further, it is not terribly uncommon to see at least some health and medical personnel pursue technical mastery with such voracity that it supplants rather than complements attentiveness to patients. Workplace familiarity also can be detrimental as, in some cases, it can blind staff members to institutional oversights and inadequacies (e.g., worn waiting room furniture, poor directional signage, excessive wait times) which are readily apparent to patients and other guests of healthcare establishments but remain unseen and unaddressed by those in positions to effect remedies [9, 21–23].

Despite good intentions, the challenges and imperfections of work life sometimes can result in inattentiveness to patients; a loss of focus that lulls employees away from mindsets of selfless service, compromising the quality of patient experiences. No one is exempt from the risks of complacency developing in their work habits. It can happen to anyone—physicians, nurses, administrators, technicians, and so on—necessitating that each and every staff member, regardless of capacity, be aware of this potential and remain on guard. While it is incumbent on individuals themselves to avoid attitudes and behaviors that diminish their sensitivities to patients, healthcare institutions must assist them in this process at every possible opportunity. Typical mechanisms used to foster patient attentiveness in workforces include employee onboarding and orientation programs, staff development initiatives, and related constructive opportunities [24–26]. These pursuits are designed to educate and enlighten staff members regarding institutional missions, their respective roles and associated importance, and the imperative of being attentive to and respectful of patients as they go about conducting their assigned duties and responsibilities.

But static initiatives, while holding immense value, have limitations, especially regarding their ability to thwart complacency that can creep into the work lives of otherwise well-intentioned personnel. What actually is needed is an ongoing dialogue between and among staff members which emphasizes customer-oriented conduct, and just as importantly, issues appropriate guidance to help personnel remain focused on this goal. One particularly effective method for encouraging patient attentiveness and a resulting customer orientation also happens to be among the simplest; a reflective exercise that encourages staff members to see themselves and their actions from the perspective of patients.

It might sound unusual to focus attention on something so seemingly rudimentary, however, seeing things from the perspective of others is not a natural process; individuals are programmed to view the world from their own eyes. But in order for personnel to be their best and deliver the most satisfying patient experiences, these self-serving instincts must be abandoned, shifting perspectives from oneself to those one serves. This necessitates that staff members be reminded to view themselves and their actions with outside eyes, ensuring the proliferation of selfless rather than selfish mindsets. To the conscientious employee, the question, “Am I seeing things through the eyes of patients?” serves as an effective reminder of priorities. It challenges staff members to critically evaluate their own actions from the viewpoint of patients, building empathy and motivating them to continually deliver their very best [27].

This reflective exercise, of course, applies to everyone working in healthcare institutions, as they all influence patients in one way or another. Physicians, nurses, and other caregivers have obvious interactions with patients making reflecting on their actions from the perspective of care recipients quite easy. How might the caregivers themselves feel on the receiving end of their own actions? Contemplating their conduct from the patient’s viewpoint can shed significant light on their behaviors, guiding improvements when shortcomings are noted. Many employees, however, do not have such prominent interactions with patients, yet their conduct influences and impacts care recipients just the same, as associated service failures illustrate.

A dietary aide, for example, who haphazardly prepares a patient’s meal has a direct hand in the diminished patient experience even without formal patient contact. A maintenance technician who does not respond in a timely fashion to repair a leaky faucet in a patient’s room certainly is not seeing things through the eyes of the care recipient. An administrator who fails to act on suggested improvements noted in a recent patient satisfaction survey also is not viewing matters from the perspective of those served. Serious infractions, of course, are resolved through disciplinary processes. This reflective exercise reaches out to those staff members who actually do care and are committed to service; it endeavors to help them ensure that their focus on patients is not lost in the rush of work life and all of the challenges that it imposes.

As for timing and placement, an introductory event can be helpful in initiating such pursuits, but vitally, an ongoing dialogue over time is required for the effort to have any lasting impact. Consider this to be an enduring conversation between and among staff members. Since viewing things from the perspective of others goes against natural tendencies, repetition is essential. Therefore, it is advised that these exercises be delivered as best practice reminders across multiple events over time within healthcare institutions. Treated in such a way, exercises can be inserted unobtrusively in virtually any context (e.g., orientation sessions, department meetings, celebratory events, etc.), requiring only a few minutes of time for delivery. Brevity facilitates routine conveyance and helps to ensure continuation, fostering all-important repetition.

Content presentation options abound, subject to the tastes and preferences of given healthcare institutions, but a suggested approach entails the following.

- Provide a succinct overview profiling the institution’s mission, the importance of operating in a customer-oriented manner, and the critical roles played by staff members in addressing the wants and needs of patients capably and respectfully.
- Compliment staff members on their dedication and commitment to the institution and to patient care and encourage them to continually deliver their very best.
- Acknowledge the complexities of work life and advise staff members to take a proactive stance against the onset of complacency which can enter subtly if one is not vigilant.
- Challenge staff members as they go about their work duties to view themselves and their actions from the perspective of patients; encourage them to think deeply to assume the role of the patient and evaluate how they would feel on the receiving end of their own efforts; and urge them to take corrective actions, as warranted, to ensure patient satisfaction.
- Conclude the session by encouraging staff members to continually ask themselves the question, “Am I seeing things through the eyes of patients?” as they go about completing their work assignments, reminding them to always view their actions from the perspective of those they serve.

For added value, products (e.g., pens, posters, magnets) featuring the “Am I seeing things through the eyes of patients?” inquiry can be circulated to staff members, helping to reinforce prior learning experiences. Sticky notes printed with the associated language are particularly effective, as they can be placed on computer monitors, bulletin boards, and other areas providing handy reminders as one completes their work. With dedicated conveyance efforts, top-of-mind awareness will result, guiding the perspectives of staff members even in the absence of direct prompts, becoming part of the cultural fabric of the institution and increasing the potential for consistent patient attention and concern. Ultimately, the ongoing dialogue supplied by the “Am I seeing things through the eyes of patients?” reflective exercise assists staff members in developing and maintaining a mindset of compassion, something quite fitting for those serving in health and medical organizations. In its highest state, true customer ambassadorship on the part of staff members emerges, benefiting personnel, institutions, and most importantly, patients.

## Conclusions

Viewing one’s actions from the perspective of patients can be very revealing, opening eyes wide and permitting opportunities for any necessary improvements, making for a simple but powerful learning experience. The “Am I seeing things through the eyes of patients?” reflective exercise helps well-intentioned staff members avoid tendencies which can lull them into states of complacency, ensuring that they remain focused on those in their care. Combined with institutional investments in related tools and techniques, a dedicated workforce committed to meeting and exceeding the wants and needs of patients affords opportunities for healthcare establishments to achieve a customer orientation, resulting in satisfied patients, loyalty, and the potential for word-of-mouth referrals which can generate additional patient volume, fostering growth and prosperity. A simple reflective exercise can go a long way toward keeping staff members on point, attentive and empathetic in service to others.
